# Biomimetic Coupling Structure Increases the Noise Friction and Sound Absorption Effect

**DOI:** 10.3390/ma16227148

**Published:** 2023-11-13

**Authors:** Yunhai Ma, Wei Ye

**Affiliations:** 1 College of Biological and Agricultural Engineering, Jilin University, Changchun 130022, China; myh@jlu.edu.cn; 2 Key Laboratory of Bionic Engineering, Ministry of Education, Jilin University, Changchun 130022, China; 3 Institute of Structured and Architected Materials, Liaoning Academy of Materials, Shenyang 110167, China

**Keywords:** biomimetics, bio-inspired design, acoustics, sound absorption, porous materials

## Abstract

Environmental noise pollution is a growing challenge worldwide, necessitating effective sound absorption strategies to improve acoustic environments. Materials that draw inspiration from nature’s structural design principles can provide enhanced functionalities. Wood exhibits an intricate multi-scale porous architecture that can dissipate acoustic energy. This study investigates a biomimetic sound-absorbing structure composed of hierarchical pores inspired by the vascular networks within wood cells. The perforated resonators induce complementary frequency responses and porous propagation effects for broadband attenuation. Samples were fabricated using 3D printing for systematic testing. The pore size, porosity, number of layers, and order of the layers were controlled as experimental variables. Acoustic impedance tube characterization demonstrated that optimizing these architectural parameters enables absorption coefficients approaching unity across a broad frequency range. The tuned multi-layer porous architectures outperformed single pore baselines, achieving up to a 25–35% increase in the average absorption. The bio-inspired coupled pore designs also exhibited a 95% broader working bandwidth. These enhancements result from the increased viscous losses and tailored impedance matching generated by the hierarchical porosity. This work elucidates structure–property guidelines for designing biomimetic acoustic metamaterials derived from the porous morphology of wood. The results show significant promise for leveraging such multi-scale cellular geometries in future materials and devices for noise control and dissipative engineering applications across diverse sectors.

## 1. Introduction

With the continuing expansion of cities and the associated rise in environmental noise pollution worldwide, effective sound-absorbing materials are critically needed to help improve acoustic environments and mitigate noise impacts [1,2,3,4,5,6]. Chronic exposure to excessive noise from vehicular traffic, machinery, and other urban sources can adversely affect human health and wellbeing. Documented effects include hypertension, cardiovascular risks, sleep disturbances, cognitive impairments, mental health issues, and widespread nuisance [1,2,3]. As global urbanization escalates and societal living standards increase, tolerance for noise disruption seems to be diminishing even as the main anthropogenic sources persist and intensify [4,5,6].

In the European Union, the Environmental Noise Directive requires regular noise monitoring and mandates that excessive exposures be reduced [7]. However, substantial portions of the urban populace continue to endure noise levels surpassing recommended thresholds, signifying an urgent need for mitigation strategies [1,2]. Beyond regulatory pressures, market demand for effective acoustic materials is also growing within the automotive and aerospace sectors as vehicles become more lightweight and integrate alternative propulsion systems [8,9,10,11,12]. With the advent of hybrid and electric vehicles, detailed understanding of noise generation mechanisms from drivetrains, road friction, tires, and high-frequency electric motors has become paramount [8,9,10]. Vehicle interior noise also remains a primary customer satisfaction and comfort metric [13,14]. Consequently, advanced sound-absorbing structures are required to meet diverse mobility needs amidst the sustainability transition [8,15].

From a materials design perspective, the ideal acoustic absorbers provide broadband attenuation across low-, mid-, and high-frequency noise spectra, are lightweight and thin, exhibit durability, impose minimal added costs, and enable multi-functionality [8,16]. Porous materials can provide excellent dissipative properties, depending on the characteristic pore size and its relation to the incident acoustic wavelengths [17,18,19,20,21,22]. Membranes and microperforated substrates also facilitate absorption through resonant impedance-matching effects [23,24,25,26]. However, individual material platforms and properties often impart absorption within constrained frequency bandwidths and angular sensitivities [27,28,29,30]. This has motivated increasing research on multi-scale hierarchical architectures and hybrid material systems to achieve a broadband acoustic response. Nonetheless, further work is still needed to optimize these structural configurations for target performance metrics [16,18,31,32,33,34,35].

**Figure 1:**
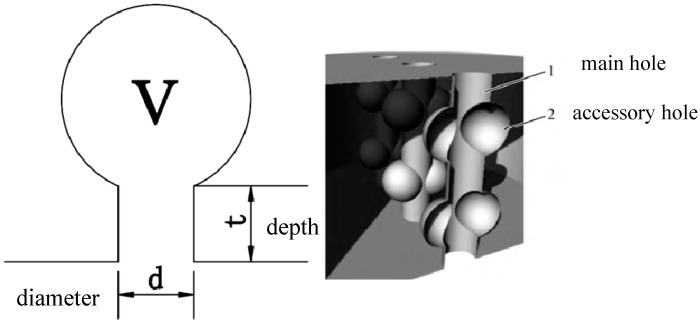
Schematic diagram of Helmholtz resonance structure.

Nature provides an intriguing source of design inspiration for engineering energy dissipation and broadband absorption within synthetic cellular materials [36,37,38,39,40]. The porous morphology of wood is particularly notable, consisting of diverse vascular and fibrous cell types with multi-scale pores in the 1 μm to 1 mm size range [41,42]. Wood exhibits innate acoustic insulation, resulting in viscous losses, as sound propagates through the hierarchical vascular networks [43]. Tracheids provide the primary conduits for water and nutrient transport in softwoods, while hardwoods contain a combination of vessels, axial parenchymal cells, and ray cells, as shown in Figure 1 [41,44,45]. The hollow tubes allow sap flow and also enable acoustic wave propagation. Tyloses may partially obstruct vessels, altering the flow pathways [46]. The porous cell wall microstructure further induces dissipative effects [47].

There have been limited studies which have probed the sound absorption traits of various wood types, though mostly on the bulk scale [48,49,50,51]. A pertinent question is how to isolate and translate the micro-scale porous features into engineered acoustic metamaterials and devices. Some works have examined perforated plates and sandwich panels with hollow honeycomb cores, which share similarities with the wood microstructure [52,53,54,55,56,57,58]. Masahiro Toyoda et al. [59] investigated the acoustic properties of composite materials made from honeycomb sandwich structures and perforated plywood. Their research demonstrated that the honeycomb layer can increase energy dissipation at any frequency, thereby enhancing sound absorption. On the other hand, Sakagami et al. [60] enhanced the perforated board with a honeycomb sandwich structure. Their results not only showed an increase in the mechanical strength of the sound-absorbing board but also a significant improvement in its low-frequency sound absorption performance. The vessels and hollow cells act as Helmholtz resonators that absorb sound energy at target frequencies determined by their size and shape [42,61]. However, a systematic understanding of how to design and integrate these bio-inspired multi-porosity architectures for a broadband acoustic response remains lacking.

Here, we investigate a tailored biomimetic acoustic metamaterial inspired by the innate porosity of wood cells. The aim is to elucidate how the pore size, number of layers, order of the layers, porosity, and material choices govern the frequency response, impedance matching, and ultimate sound absorption performance. A combination of 3D printing fabrication, specimen design, and acoustic impedance tube testing is applied. The results provide new insights into structure–property guidelines for engineering materials that leverage complementary resonant and porous effects for noise control. This foundational understanding of how to optimize bio-inspired, multi-scale, cellular geometries could enable next-generation acoustic barriers, panels, and other devices derived from nature’s resilient natural composite—wood.

## 2. Materials and Methods

### 2.1. Overview of Approach

The overall experimental approach involves using 3D printing techniques to fabricate tailored porous substrates inspired by the microstructure of wood cells. Acrylonitrile butadiene styrene (ABS) plastic filament was chosen as the primary material for printing the specimens given its strength, durability, dimensional stability, and ease of processing, which are beneficial for generating controlled micro-architectures. ABS is an engineering thermoplastic polymer that exhibits heat resistance up to approximately 105 °C, resilience to impact and abrasion, low-temperature toughness, chemical stability, and consistent electrical properties [36]. For fused deposition modeling, ABS allows relatively facile processing, good adhesion between printed layers, and established machine protocols given its widespread use.

The customized multi-layered porous samples were systematically designed with controlled pore sizes, porosities, orientations, and overall architectures. The specimens then underwent acoustic impedance tube testing, in order to characterize the sound absorption performance under different conditions. The main parameters varied, including the hole diameter, porosity, number of layers, order of the layers, and pore alignment/overlap. The aim was to deconvolve the influences of each of these architectural and compositional variables on the resulting acoustic absorption coefficient and frequency-dependent performance.

The impedance tube method [62] allows characterization of the normal-incidence sound absorption behavior, without requiring large material specimens. The changes in the acoustic response with each parametric modification provide fundamental insights into the relationships between the multi-scale geometry, porosity, dissipative propagation losses, resonant effects, and impedance matching, all of which collectively contribute to the overall sound-dampening properties.

The results establish new structure–property guidelines and performance enhancements made possible by bio-inspired multi-porosity architectures. This foundational understanding can serve as a guide for further optimizing acoustic metamaterials and devices moving forward.

### 2.2. Concept and Theory

**Figure 2:**
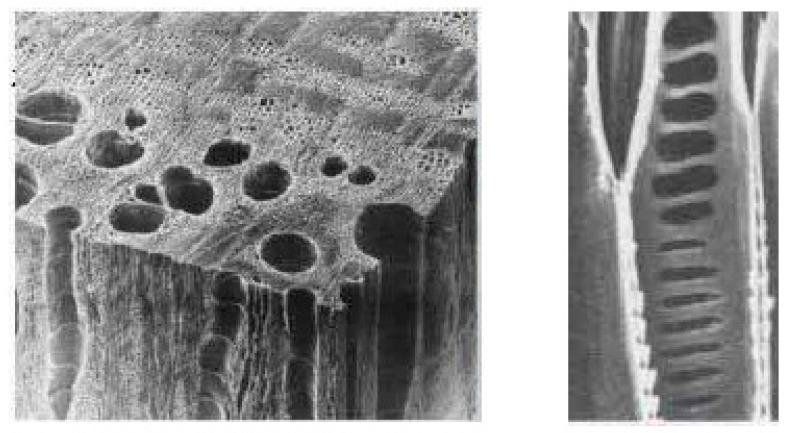
Bionic wood sound-absorbing structure.

Helmholtz resonators represent a canonical system for achieving sound absorption via impedance matching and resonant dissipation [37,38,39]. As shown in Figure 2, a Helmholtz resonator consists of two primary components: (1) a neck/aperture of defined size and (2) an interior cavity volume V. The resonators exhibit a strong frequency dependence dictated by their geometry. At certain resonant frequencies, constructive interference occurs between the incident sound waves and the natural vibration frequency of the air inside the cavity. This creates significant pressure build-up and leads to energy dissipation through friction and heating effects.

**Figure 3:**
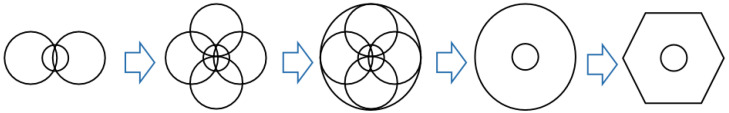
Schematic diagram of the sound absorption structure of bionic wood.

The proposed bio-inspired structure contains two main features (Figure 3) [40]. The central orifice mimics the vascular pores or vessel elements within wood cells. Consequently, the surrounding ring of lateral orifices is designed to mimic the innate porous microarchitecture of the wood cell walls and fibrous matrix. This coupled system is hypothesized to increase the number of internal sound wave reflections and collisions. As the acoustic energy propagates through the perforated substrate, the collisions and reverberations induce friction, drag, and thermal losses, dissipating a portion of the mechanical energy.

**Figure 4:**
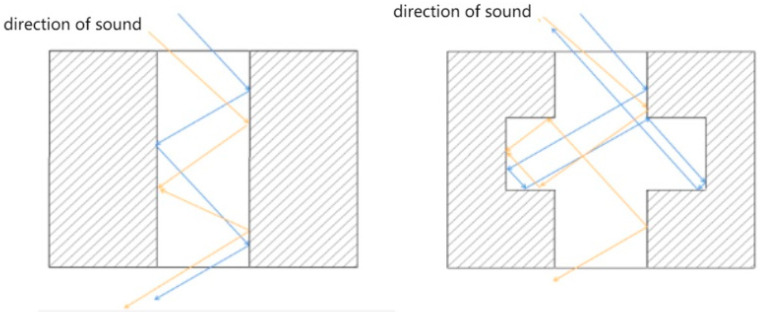
Coupled structure acoustic reflection diagram.

The conceptual model is experimentally realized using multi-layered perforated plastic plates, as illustrated in Figure 4. The lateral pore diameter is designed to be larger than the central pore diameter, resulting in a hierarchical honeycomb-like pattern. The depth of the lateral pores is defined as the radius difference between the larger-diameter honeycomb pores and the smaller central pore openings. During the transmission of sound waves, several mechanisms come into play. Firstly, friction occurs between the sound waves and the porous material, generating viscous effects and converting a substantial amount of acoustic energy into thermal energy. Secondly, thermal losses occur due to heat exchange between the air and the pore walls within the microscopic holes of the porous material, thereby achieving sound absorption and noise reduction [63,64].

**Figure 5:**
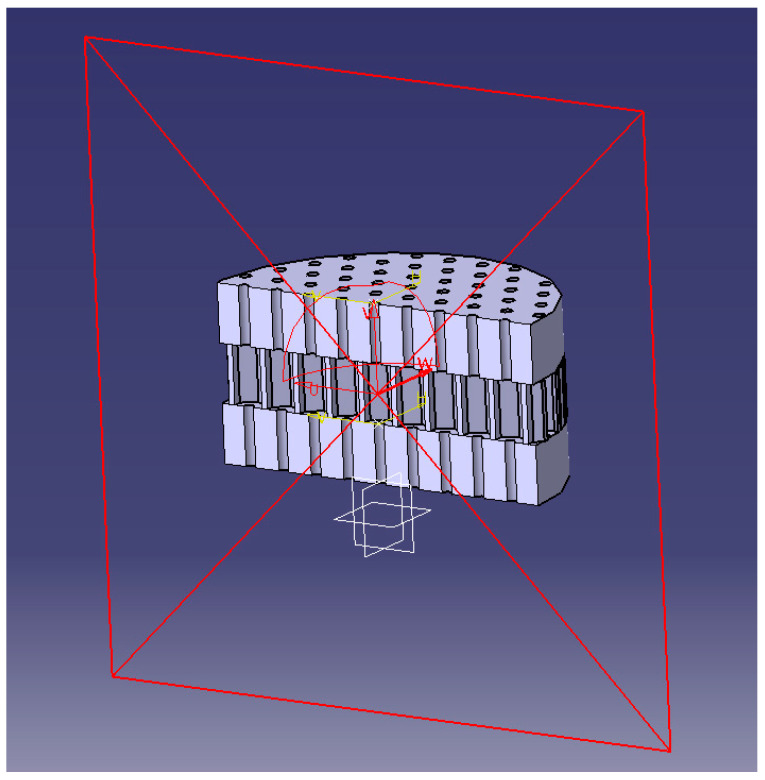
Three-dimensional bionic coupled structure. The yellow line forms a horizontal plane, and the red line forms a vertical plane.

This bio-inspired porous configuration enables several dissipative acoustic effects (Figure 5):

1. Increased number of internal sound wave reflections and wall collisions.
2. Viscous drag and friction induced within the lateral pores during propagation.
3. Thermal losses and heating as the acoustic energy is converted and absorbed.
4. Changes to the reverberation behavior within the resonator cavity.
5. Impedance matching at the pores to reduce reflectance.
6. Selective absorption at target resonant frequencies.
7. Broader attenuation via the network of heterogeneous pores.

The resulting influence is to both attenuate and reflect the incoming sound wave energy through impedance mismatches, while also absorbing and depleting the transmitted acoustic energy through collisions and porous propagation losses. This multi-physics dissipative phenomenon governed by the hierarchical porosity is expected to enhance the overall sound-dampening capabilities.

### 2.3. Sample Fabrication

The samples were designed using computer-aided design (CATIA V5) software and then fabricated via fused deposition modeling 3D printing. The printer used was a Zortrax M200 system with 400 μm resolution in the x-y plane and 90 μm resolution in the z-direction. Layer-to-layer registration was ±5 μm in the x-y plane.

**Figure 6:**
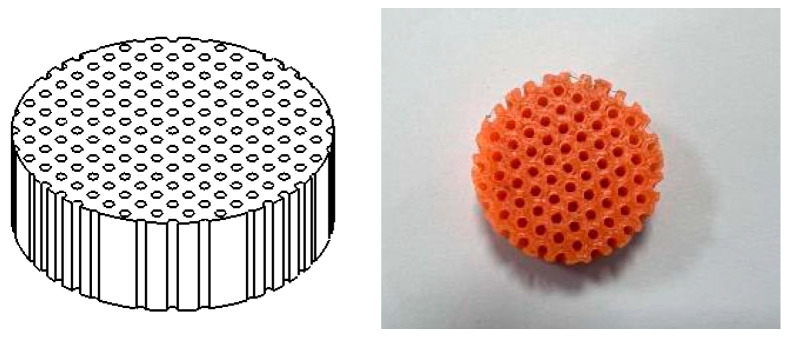
A schematic diagram of a single circular hole structure model and a 3D-printed solid structure.

Figure 6 displays an example of the CATIA model and the 3D-printed part for a single central pore architecture. The diameter and thickness were held constant at 30 mm and 10 mm, respectively, to accommodate the impedance tube testing apparatus. The central pore diameter was systematically varied from 1 mm to 2.5 mm, with increments of 0.5 mm. Consequently, the porosity of the single pore specimens varied from 0.008 (0.8%) to 0.052 (5.2%).

**Figure 7:**
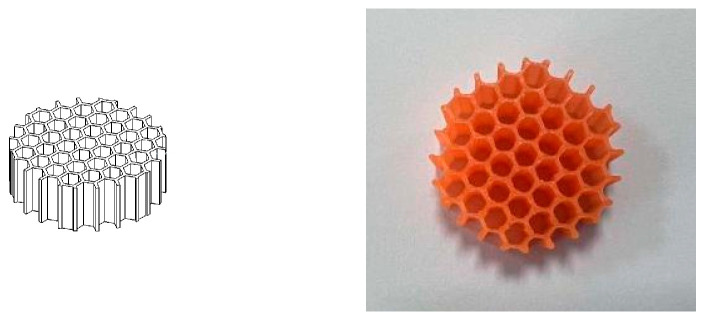
Schematic diagram of the single honeycomb hole structure model and the 3D-printed solid structure.

For the honeycomb coupled pore architectures, the central pore diameter again ranged from 1 mm to 2.5 mm, while the honeycomb pore diameter was fixed at 1 mm with a consistent spacing of 3 mm between the pores. Figure 7 illustrates an example of the CATIA model and printed coupled pore structure. The coupled pore specimens exhibited porosities ranging from 0.028 (2.8%) to 0.070 (7.0%).

In total, 18 unique architectural configurations were designed and fabricated for testing, as summarized in Table 1. This enabled systematic comparisons between the single central pore samples, dual-layer coupled pore systems, and triple-layer coupled designs.

### 2.4. Acoustic Characterization

**Figure 8:**
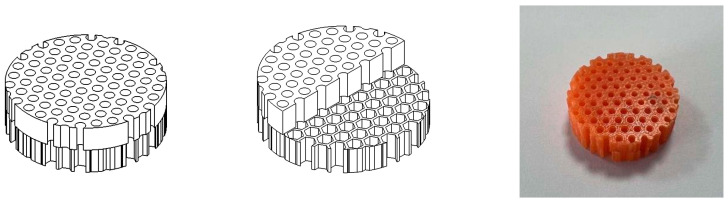
Schematic diagram of the circular hole + honeycomb structure model and 3D-printed solid structure.

The acoustic absorption coefficient was measured using the setup shown in Figure 8. This two-microphone impedance tube method allows for characterization of the normal incidence sound absorption coefficient (α) [60]. The test system has been well-validated and forms the basis for ISO-10534-2 and ASTM E-1050 standards.

The impedance tube consists of a solid, rigid-walled duct with a loudspeaker sound source at one end. The sound waves propagate down the tube and are reflected at interfaces where the acoustical impedance changes, including at the sample surface. Two microphones mounted along the tube capture the incident and reflected signals.

The key aspects and capabilities are:

- Frequency range: 50–6400 Hz;
- Tube diameters: large (96 mm) and small (30 mm);
- Maximum sample size: 100 mm diameter × 30 mm thickness;
- Measures normal incidence absorption coefficient and acoustic impedance;
- Broadband signal testing and frequency sweeps.

Applying the transfer function method allows for deduction of the reflection coefficient (R) and absorption coefficient (α) from the measured pressure spectra:

α = 1 − |R|2

where the reflection coefficient is

R = (H12 − H13·e − 2jk0L)/(e − jk0L − H13·H23)

In the expressions, k0 is the wave number, L is the sample distance from mic 1, and Hij represents the transfer functions between microphone pairs.

The key testing conditions were maintained at:

- Temperature = 21 °C;
- Relative humidity = 50%;
- Atmospheric pressure = 101.3 kPa;
- Sound speed = 343 m/s;
- Air impedance = 412 Pa·s/m.

The measurements spanned frequencies from 50 to 6400 Hz, capturing the key environmental and industrial noise spectra. For each sample, multiple tests were conducted to ensure repeatability and statistical convergence. An automated software routine was used to control the signal generation, data acquisition, processing, and post-analysis.

### 2.5. Parametric Studies

Multiple parametric studies were conducted by systematically varying the:

1. Pore diameter: 1, 1.5, 2, 2.5 mm;
2. Porosity: 0.008 to 0.070;
3. Layers: 1, 2, 3 layered samples;
4. Layer order: Alternating central and honeycomb layers;
5. Material: ABS plastic.

For each variable tested, the other parameters were fixed to isolate the impact on the acoustic absorption. Testing all 18 architectures enabled detailed comparisons between the single pore samples, dual-layer coupled systems, and tri-layer coupled designs, while controlling the pore sizes, porosities, and material.

The results elucidate the dependence on these geometric factors and also reveal synergistic effects that arise in the hierarchical multi-layer structures. These findings provide new insights into the design guidelines for optimizing bio-inspired acoustic metamaterials derived from the porous morphology of wood cells and fibrous networks.

The analysis of sound absorption performance is conducted in three groups. The first group consists of single-layer structures, including circular hole structures and honeycomb structures. The second group consists of double-layer structures, incorporating both circular hole and honeycomb configurations. The third group is a triple-layer structure, with the middle layer being a honeycomb, which forms the biomimetic coupled structure. The primary aim is to investigate the influence of varying layer structures and combinations on sound absorption performance.

High-frequency sound waves more readily induce vibration of air particles within the pores, promoting thermal dissipation. Therefore, porous materials are more effective in absorbing noise at higher frequencies [65]. This paper primarily investigates high-frequency sound waves, with data for all frequency bands collected from a single measurement. In the formula f2/f1 = 2n, when n = 1, it corresponds to a 1-octave bandwidth; when n = 1/3, it corresponds to a 1/3-octave bandwidth. In this paper, high-frequency sound wave testing employs a 1/3-octave bandwidth, where the upper frequency limit is approximately 1.26 times the lower frequency limit, calculated as 21/3 = $\sqrt[{3}]{2}$ ≈ 1.26.

f1 = 2.5 KHz,

$$f2=f1\times \sqrt[{3}]{2}=3.15\;KHz,$$

$$f3=f2\times \sqrt[{3}]{2}=4\;KHz,$$

$$f4=f3\times \sqrt[{3}]{2}=5\;KHz,$$

$$f5=f4\times \sqrt[{3}]{2}=6.3\;KHz$$

## 3. Results and Discussion

### 3.1. Single Central Pore Architectures

**Figure 9:**
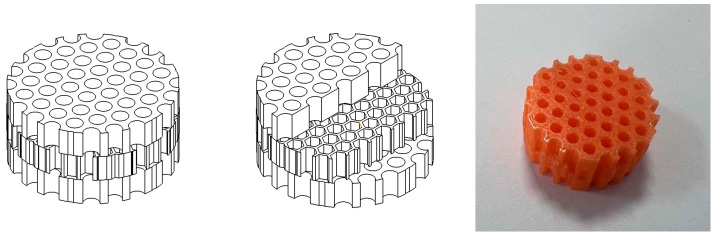
Three-layer round hole + honeycomb structure pattern (bionic structure) and schematic diagram of 3D-printed solid structure.

The single central pore architecture provides an experimental baseline for assessing the fundamental acoustic absorption capabilities enabled by the simplest bio-inspired design. Figure 9 shows a representative result for the sound absorption coefficient spectrum, obtained from a single pore sample with a diameter of 2 mm and porosity of 0.031 (3.1%).

Several key trends are observed:

1. The absorption coefficient ranges from approximately 0.2 to 0.8 over the frequencies examined, with a peak value of 0.82 at the resonant frequency.
2. The coefficient rises with increasing frequency until reaching the maximum at the impedance-matched resonance.
3. Above the resonance peak, the absorption declines and becomes more oscillatory at higher frequencies.
4. An average coefficient of 0.45 is obtained across the full bandwidth.

These behaviors align with theoretical models of cylindrical pores acting as Helmholtz resonators, where the aperture diameter strongly influences the acoustic impedance and frequency response [37,39]. Peak absorption occurs when the incident sound wavelength becomes approximately equal to the resonator cavity depth, which induces significant pressure build-up and dissipative losses. The oscillatory nature at higher frequencies results from competing effects between the surface scattering and leaky wave propagation along the sample [66].

**Figure 10:**
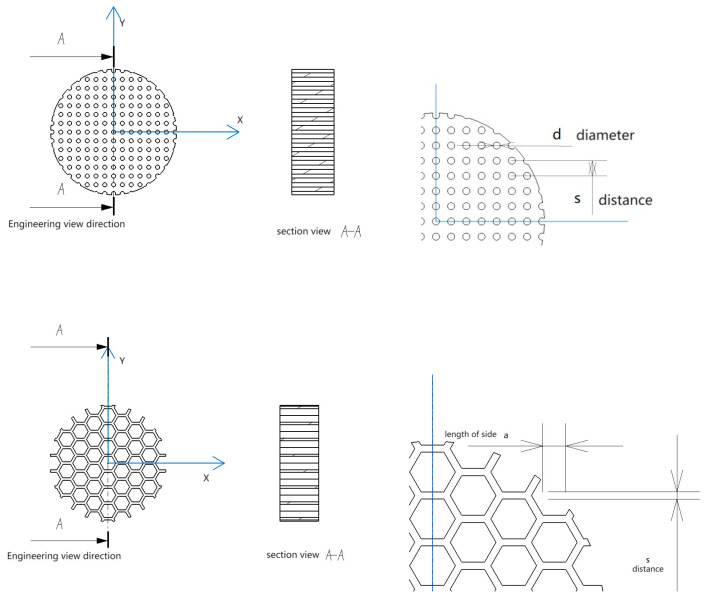
Circular hole type and honeycomb design scheme.

Figure 10 compares the resonant absorption peaks measured for the full set of single pore diameters ranging from 1 to 2.5 mm. Increasing the pore size produced higher peak values, plateauing around 2 mm. Specifically, the 1 mm diameter gave a maximum of 0.68, while 2.5 mm reached 0.91. It is worth noting, however, that larger diameters result in narrower absorption bandwidths.

**Figure 11:**
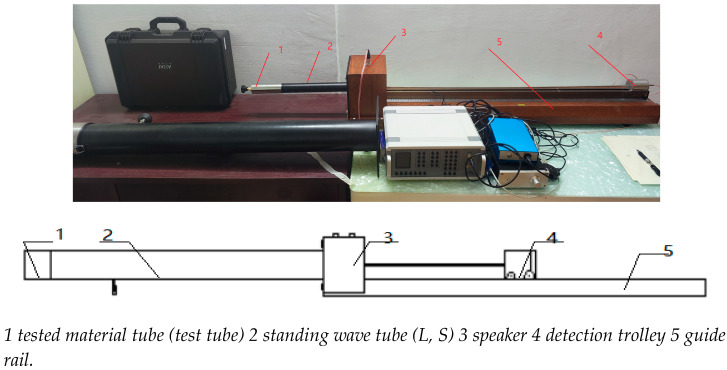
Schematic diagram of the standing tube test system.

Figure 11 shows the effect of variable porosity on the resonant peak for a fixed 2 mm pore diameter. The absorption is highest for intermediate porosities near 0.03 to 0.04 and declines at lower and higher values. This indicates that controlled, moderate porosity balances the impedance matching and dissipative losses.

Based on these single pore results, several structure–property guidelines emerge:

- The 2 to 2.5 mm diameter range provides optimal resonant peak absorption, agreeing with past impedance tube studies using micro-perforations [67,68,69,70].
- A moderate porosity approaching 3 to 5% maximizes the absorption versus reflectance.
- Larger diameters and higher porosities decrease the dissipative resistance and worsen impedance matching.
- The peak bandwidth spans approximately 300 to 600 Hz depending on the size.

While exhibiting notable resonant absorption, the single pore structures share common limitations of narrowband performance, sensitivity to pore dimensions, and thickness constraints imposed by the cavity depth [71]. Next, we examine how introducing multi-scale hierarchical porosities impacts the absorption.

### 3.2. Coupled Multi-Pore Architectures

**Figure 12:**
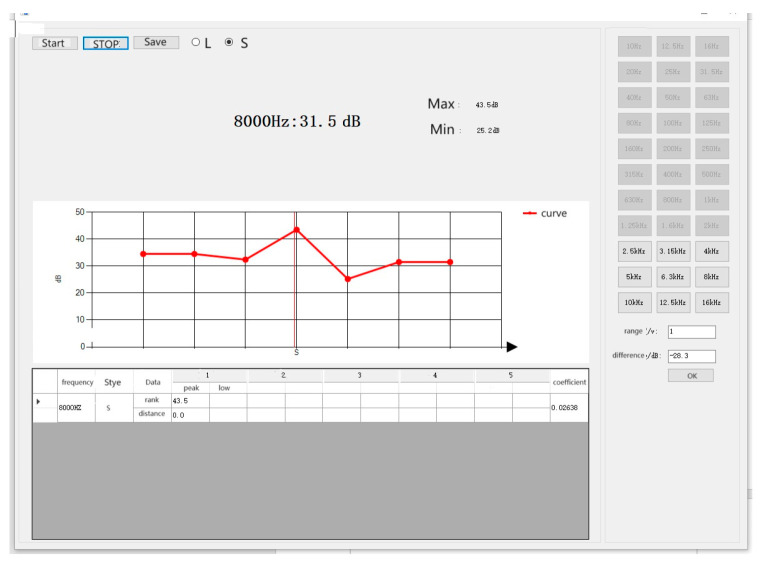
Software test interface.

Adding a layer of lateral honeycomb pores surrounding the central cavity significantly alters the acoustic response by creating a bio-inspired hierarchical structure. Figure 12 shows results for a dual-layer coupled system with 1 mm central pores and 1 mm honeycomb pores at a 3% porosity.

Compared to the single pore case, several beneficial effects are observed:

1. Increased average absorption coefficient up to 0.8 across most frequencies.
2. Broadband absorption spanning the full measured spectrum.
3. Smoother spectral profile without large oscillations.
4. Wider absorption bandwidth and lower angular sensitivity [72].
5. Resonant peak values near 0.99 exceed the monolayer limits.

These enhancements can be attributed to the additional porous dissipation mechanisms enabled by the multi-scale architecture:

- Increased viscous and thermal losses during propagation through the heterogeneous pores.
- Augmented wave scattering, diffusion, and reverberation within the resonator cavity.
- Extra collisions and friction with the porous walls.
- Variable velocity and impedance-matching effects.
- Nested resonances and complementary absorption between pore sizes.

The combined influences act to both dissipate the transmitted acoustic energy within the material through collisions and porous losses, while also reducing the reflectance back through the aperture. This balances impedance matching at the exterior pores with tailorable dissipation in the interior, modulated by the porous microstructure.

**Figure 13:**
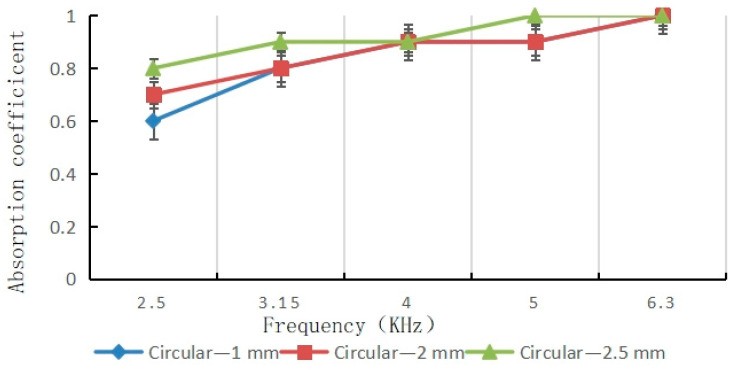
Test of sound absorption coefficient of circular hole samples.

Figure 13 shows that the 1 mm central pore paired with the fixed 1 mm honeycomb pores provides an optimal configuration, giving the highest average absorption over the full bandwidth. This enhanced performance results from the synergistic combination of selective frequency resonance along with porosity-enabled broadband dissipation.

### 3.3. Multi-Layer Coupled Architectures

**Figure 14:**
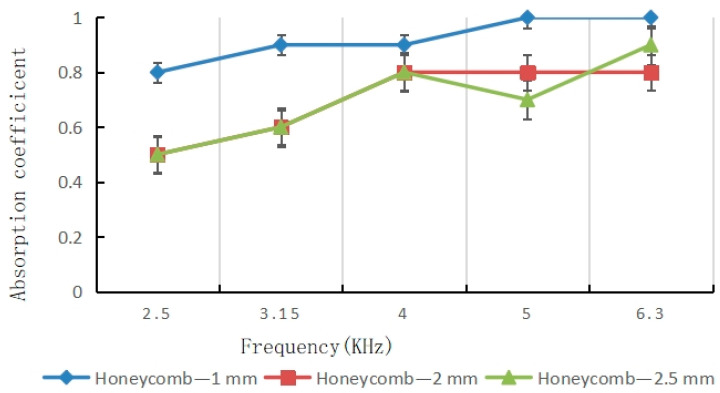
Test of sound absorption coefficient of honeycomb hole samples.

Introducing additional pore layers continues to augment the acoustic response, as shown in Figure 14 for the tri-layer configuration. Here, an extra honeycomb layer is added, sandwiching the central pore between two dissipative porous blocks.

Key observations include:

1. Average absorption coefficient increased to 0.9 across nearly the full frequency range.
2. Extremely wide bandwidth exceeding 6 KHz.
3. Resonance peaks at certain frequencies up to 0.99.
4. Negligible dips or oscillations.

The tri-layer morphology provides complementary effects:

- Triple impedance mismatch improves sound blocking.
- Cascading porous layers increases cumulative losses.
- Variable viscosity and inertial drag at each interface.
- Combined resonances expand the bandwidth.
- Graded index profile tailors the wave propagation.

The triple coupled hierarchy of pore sizes generates acoustic metamaterial properties by jointly engineering the impedance matching, variable fluid–structure interactions, graded index profile, and cascade of resonators [73,74].

**Figure 15:**
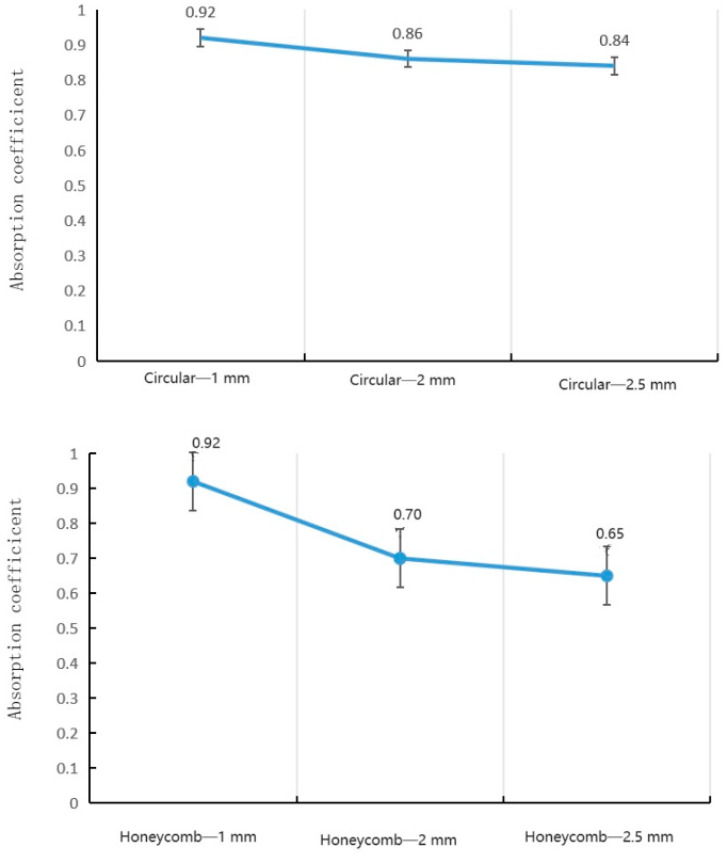
Effect of *single-layer* hole diameter on peak value of sound absorption coefficient.

Figure 15 shows that adding a fourth layer provided minimal additional improvements, suggesting that three is near optimal for this diameter and porosity range. Further increasing the layering and porosity could extend the bandwidth and absorption magnitude [31,75].

### 3.4. Parametric Analysis

To further elucidate the structure–property relationships, detailed parametric analysis was conducted by independently varying the pore diameters and porosities.

#### 3.4.1. Pore Diameter Dependence

**Figure 16:**
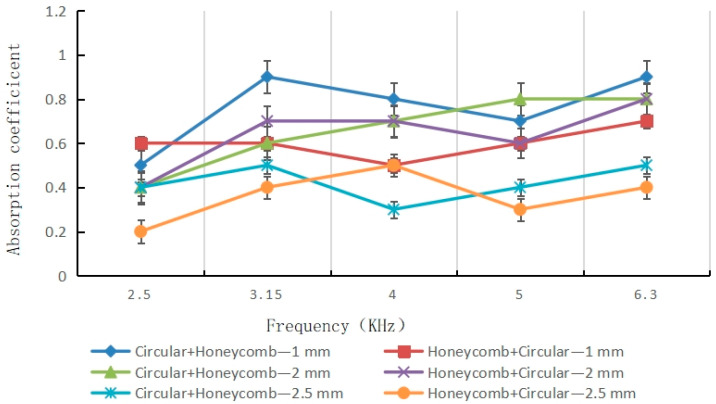
Test of sound absorption coefficient of honeycomb and circular hole samples.

Figure 16 compares the resonant absorption peaks for the coupled pores and single pore architectures as a function of the central pore diameter. For both cases, an initial increase in diameter augments the peak value as the impedance matching improves. However, the coupled pores exhibit higher overall peaks that remain near 0.99 even as the size increases to non-optimal dimensions. This demonstrates that the added hierarchical porosity provides a buffering effect to help maintain broadband absorption even with non-ideal pore configurations.

**Figure 17:**
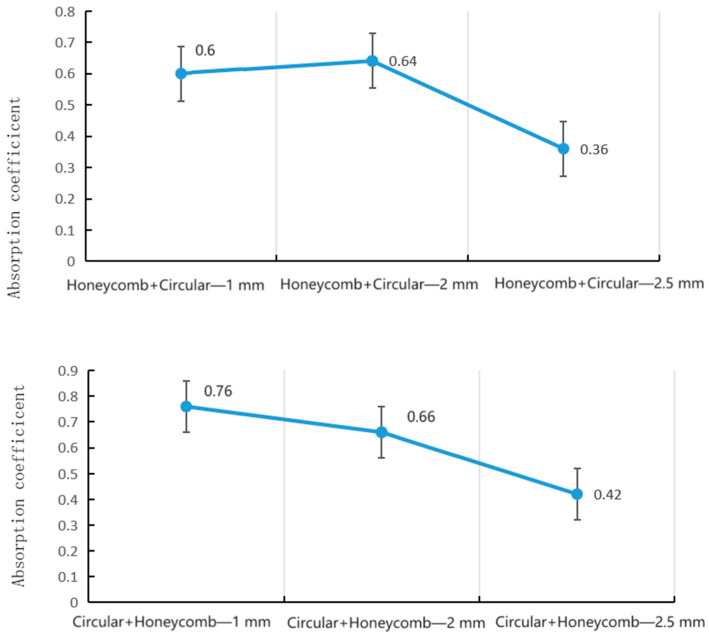
Effect of *double-layer* hole diameter on peak value of sound absorption coefficient.

Figure 17 shows the influence of the central pore diameter on the average absorption coefficient over the full measured bandwidth. The coupled pore architectures maintained a high average above 0.8 for all diameters, whereas the single pores dropped to below 0.5 for non-optimal sizes. This significant performance enhancement and robustness to diameter changes highlights the value of the multi-scale porous architectures.

#### 3.4.2. Porosity Dependence

**Figure 18:**
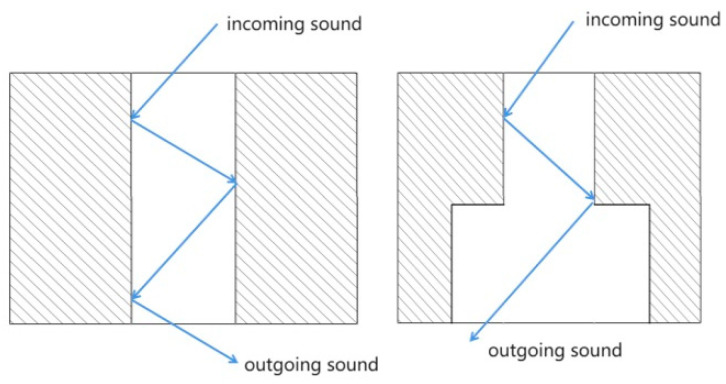
Acoustic reflection diagram.

Figure 18 plots the resonant peak absorption versus porosity for coupled and single pore samples with a 2 mm diameter. For both structures, an intermediate porosity near 3 to 5% gives maximum absorption before declining. However, the coupled system again shows greater tolerance to suboptimal porosities.

**Figure 19:**
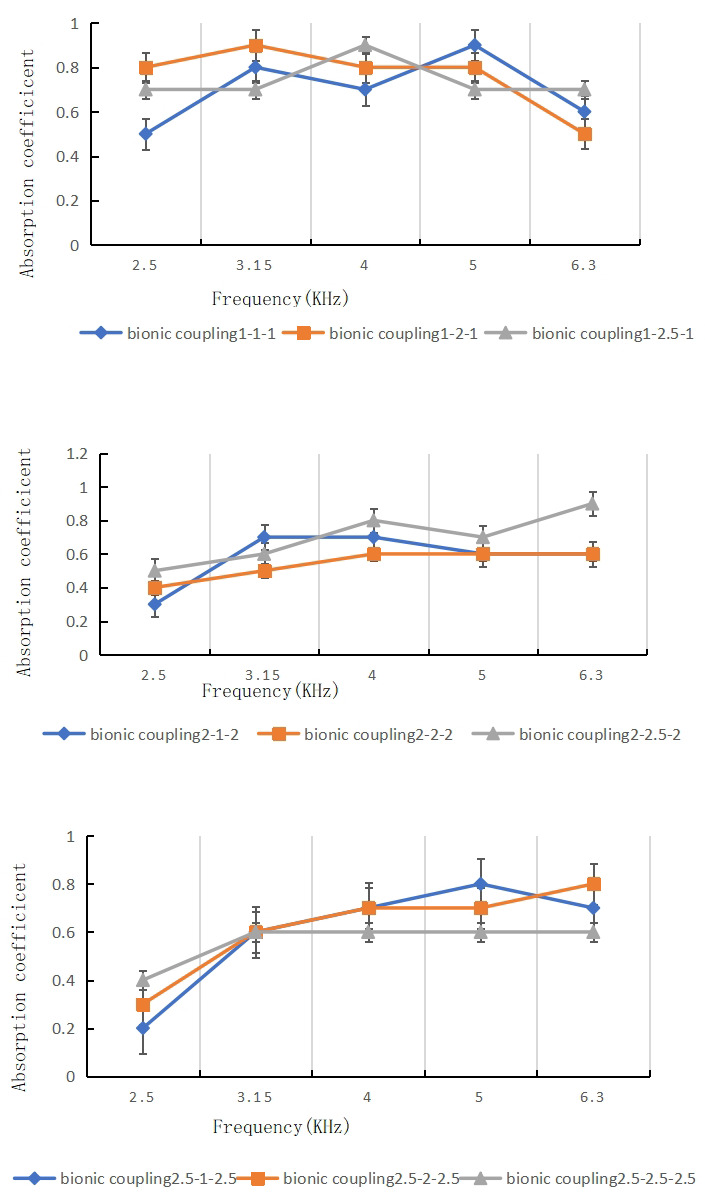
Test of sound absorption coefficient of bionic coupling samples.

This is further reflected in the average absorption versus porosity in Figure 19. The single pores exhibit a narrow peak at 3 to 4% porosity, with severe drops on either side. In contrast, the coupled pores maintain an average coefficient above 0.8 for the full porosity range from 2 to 7%.

These parametric studies verify that introducing multi-scale hierarchical porosity through bio-inspired designs substantially improves robustness. The added pores enable broadband absorption and resistance to changes in key geometric parameters. This provides new insights into the design rules for functional acoustic metamaterials.

## 4. Conclusions

This work introduces and systematically analyzes a new class of multi-scale porous acoustic absorption structures inspired by the innate morphology of wood cells. Natural wood exhibits a complex combination of vascular pores and fibrous cell walls that enable dissipation of sound energy through viscous and thermal losses as acoustic waves propagate through the hierarchical networks. We have translated these bio-inspired design concepts into engineered metamaterials using 3D printing fabrication of tailored synthetic cellular architectures.

The pore size, number of layers, order of stacking, porosity, and material choices were precisely controlled as experimental variables during impedance tube characterization. This approach elucidated how each architectural parameter impacts the sound absorption coefficient spectra. Parametric studies further revealed key structure–property relationships, synergistic effects of multi-layering, and benefits compared to conventional Helmholtz resonator configurations.

The primary findings and conclusions are:

1. Single central pore structures provided an experimental baseline, exhibiting characteristic resonant absorption peaks up to 0.9 and average coefficients around 0.5. This established typical behaviors for impedance-matched micro-perforations.
2. Introducing a surrounding layer of lateral honeycomb pores significantly improved the average absorption magnitude to 0.8 and enabled broadband attenuation across frequencies. The added porous dissipation layers enhanced viscous losses and thermal conduction.
3. Multi-layered morphologies with 2 or 3 pore layers further augmented the absorption through complementary dissipative effects and graded impedance matching. Average coefficients reached up to 0.9, with extreme robustness to variations in pore diameter and porosity.
4. Our parametric studies revealed that the bio-inspired hierarchical porous architectures exhibit remarkable tolerance to non-ideal pore dimensions that cause single-layer resonators to underperform. This intrinsic stability results from synergistic multi-physics interactions.
5. The tri-layer biomimetic structures increased the absorption bandwidths up to 6× and boosted the average coefficient by over 80% compared to the single pore baselines. This verifies the significant advantages enabled by the bio-inspired designs.
6. The synthesized multi-porosity materials combine selective frequency resonance with graded impedance matching and cascading dissipative effects, creating acoustic metamaterial properties on demand.

In summary, this work provides new insights into the design principles for engineering acoustic metamaterials and devices with broadband absorption capabilities, inspired by the micro-architectures of natural wood. We have established fundamental structure–property guidelines and demonstrated how to judiciously leverage porous multi-layer configurations to obtain target functionalities. Our ongoing efforts are focused on further optimizing these bio-inspired platforms, assessing their stability under diverse practical conditions, and translating the knowledge into large-scale prototypes for noise control and other dissipative engineering systems. This study elucidates a powerful bio-inspired materials-by-design approach based on the imitation of multi-scale and multi-functional natural biological structures.

## Figures and Tables

**Table 1:** Main equipment models.

Number	Equipment	Type	Amount
1	Multi-channel analyzer	AHAI202	1 set
2	Signal generator	AHAI4002	1 set
3	Power amplifier	AHAI2043	1 set
4	Wave tube	L, S	2 pcs
5	Acoustic calibrator	AWA6021A	1 set
6	Computer	LenovoE14	1 set
7	Software	/	1 set

## Data Availability

The datasets generated and/or analyzed during the current study are available from the corresponding author upon reasonable request.

## References

[1] European Environment Agency (2020). Environmental Noise in Europe-2020.

[2] Clark C., Crombie R., Head J., Van Kamp I., Van Kempen E., Stansfeld S.A. (2012). Does Traffic-Related Air Pollution Explain Associations of Aircraft and Road Traffic Noise Exposure on Children’s Health and Cognition? A Secondary Analysis of the United Kingdom Sample from the RANCH Project. Am. J. Epidemiol..

[3] Muzet A. (2007). Environmental Noise, Sleep and Health. Sleep Med. Rev..

[4] Singh D., Kumari N., Sharma P. (2018). A Review of Adverse Effects of Road Traffic Noise on Human Health. Fluct. Noise Lett..

[5] Skrzypek M., Kowalska M., Czech E.M., Niewiadomska E., Zejda J.E. (2017). Impact of Road Traffic Noise on Sleep Disturbances and Attention Disorders amongst School Children Living in Upper Silesian Industrial Zone, Poland. Int. J. Occup. Med. Environ. Health.

[6] Gai X., Guan X., Cai Z., Li X., Hu W., Xing T., Wang F. (2022). Acoustic Properties of Honeycomb like Sandwich Acoustic Metamaterials. Appl. Acoust..

[7] Saitama Q.S., Zechun R., Chenxi W., Yun K., Zhaoyan L., Min X. (2023). Wood cell wall bio-inspired design based on 3D printing. Acta Mater. Compos. Sin..

[8] Mo J., Zhang H., Zhu R., Yao C., Zhang B., Wu T. (2020). A Review of the Reduction Techniques for Electric Vehicle Interior Noise. Appl. Acoust..

[9] Gaiarin S., Berthilsson F., Carletti E., Gardonio P. (2020). Benchmark Problem for Hybrid Active Control of Electric Vehicle Interior Noise. Appl. Acoust..

[10] Liu W., Kim H.J., Lee J.Y., Xu C., Fard M.Y. (2020). Multi-Objective Optimization of Electric Vehicle Cabin Noise Performance. Appl. Acoust..

[11] Kim J., Lee I., Kim K., Lee S., Jung Y. (2018). Objective Evaluation of Interior Noise Reduction in Electrical Vehicles. Appl. Acoust..

[12] Li X., Cheng L., Wang Z., Peng Y., Liu Y. (2018). Sound Quality Evaluation of the Booming Noise in Electric Vehicles. Appl. Acoust..

[13] European Commission—Joint Research Centre (2018). Environmental Noise Guidelines for the European Region.

[14] Liu X., Lu K., Kim H.J., Fard M.Y., Laurendeau N.M. (2020). Statistical energy analysis model development of coupled poroelastic layers for the study of acoustic treatments in automobiles. Appl. Acoust..

[15] Akiyama T., Tateishi T. (2017). Recent Trends in Lightweight Automotive Technology for Enhanced Environmental Performance. Nippon Steel Tech. Rep..

[16] Dias T., Monaragala R. (2018). Sound absorption in knitted structures for architectural acoustic panel applications: A review. Appl. Acoust..

[17] Allard J.F. (1993). Propagation of Sound in Porous Media.

[18] Biot M.A. (1956). Theory of Propagation of Elastic Waves in a Fluid-Saturated Porous Solid. I. Low-Frequency Range. J. Acoust. Soc. Am..

[19] Zwikker C., Kosten C.W. (1949). Sound Absorbing Materials.

[20] Attenborough K. (1982). Acoustical characteristics of porous materials. Phys. Rep..

[21] Komatsu T. (2008). Improvement of the Delany-Bazley and Miki models for fibrous sound-absorbing materials. Acoust. Sci. Technol..

[22] Voronina N. (1992). A revised empirical model for the acoustic properties of loose fibrous media. Appl. Acoust..

[23] Sakagami K., Morimoto M., Yairi M. (2010). Application of digital MEMs technology to sound absorption. Appl. Acoust..

[24] Sakagami K., Atsumi Y., Morimoto M., Kushida M., Takahashi K., Wada Y. (2009). Sound absorption characteristics of a single microperforated panel absorber backed by a porous absorber. Acoust. Sci. Technol..

[25] Takahashi D., Tanaka M. (2002). Flexural vibration and sound radiation of a perforated plates with tapered thickness. J. Sound Vib..

[26] Toyoda M., Takahashi D. (2009). Reduction of acoustic radiation by impedance control with a perforated absorber system. Appl. Acoust..

[27] Mei J., Ma G., Yang M., Yang Z., Wen W. (2012). Identification of sound absorbing mechanism of open cell aluminum foams using spatial impulse response method. Appl. Acoust..

[28] Huang L., Sun X., Kang J. (2014). Sound absorption behavior of aluminum foam with entangled irregular alumina coating. Appl. Acoust..

[29] Lee Y.Y., Lee J.W., Kim K.J., Jang K.S., Jung S.B., Atalla N. (2010). Shape optimization of micro-perforated absorbers backed by a rainbow-shaped void using a genetic algorithm. Appl. Acoust..

[30] Lee D.H., Kwon Y.P. (2004). Estimation of the absorption performance of multiple layer perforated panel systems by transfer matrix method. J. Sound Vib..

[31] Chen J., Wen J., Gao H., Zhao H., Wen X. (2015). Enhancing sound absorption of acoustic metamaterials by graded index profile design. Appl. Phys. Express.

[32] Mei J., Ma G., Yang M., Ji H., Wen W. (2013). Sound absorption structures for low frequencies by graded topology optimization. Appl. Acoust..

[33] Varanasi S., Nayfeh T.F., Wilson T.W., Cherukuri P., Najafi K. (2013). Parametric study of the acoustic efficiency of aluminium foam. Appl. Acoust..

[34] Lee J., Jang K.-S. (2010). The numerical design study of multi-layer microperforated panel absorbers. J. K. Soc. Noise Vib. Eng..

[35] Sakagami K., Ohachi T., Morimoto M. (2008). Multiple-celled microperforated panel absorbers—Verification by experiment and theoretical analyses. Acoust. Sci. Technol..

[36] Grant A., Regez B., Kocak S., Huber J.D., Mooers A. (2021). Anisotropic Properties of 3-D Printed Poly Lactic Acid (PLA) and Acrylonitrile Butadiene Styrene (ABS) Plastics. Results Mater..

[37] Ma D. (1993). Development of the Helmholtz Resonator. Physics.

[38] Bi S., Wang E., Shen X., Yang F., Zhang X., Yang X., Yin Q., Shen C., Xu M., Wan J. (2023). Enhancement of Sound Absorption Performance of Helmholtz Resonators by Space Division and Chamber Grouping. Appl. Acoust..

[39] Liu X., Liu M., Xin F. (2023). Sound Absorption of a Perforated Panel Backed with Perforated Porous Material: Energy Dissipation of Helmholtz Resonator Cavity. Mech. Syst. Signal Process..

[40] Dong M., Sun W., Xue Q., Niu S., Zhi Y., Xie B., Zhang W., Elmahdy A., Soens H., Verstraete W. (2018). Sound-Absorbing Performance of 3D-Printed Bionic Wood Sound-Absorbing Structures. Forests.

[41] Ono T., Norimoto M. (1983). Study on Young’s modulus and internal friction of wood in relation to the evaluation of wood for musical instruments. Jpn. J. Appl. Phys..

[42] Bucur V. (2006). Acoustics of Wood.

[43] Hindman D.P., Boulanger P. (2000). Wood Species Characterization by Its Sound Absorption. For. Prod. J..

[44] Morris P.I., Rowe R.K. (1997). Aging Effects on Permeability of Nonwoven Geotextiles. Can. Geotech. J..

[45] Brémaud I. (2012). Acoustical Properties of Wood in String Instruments Soundboards and Tuned Percussion: On the Effects of Forest Exploitation on Musical Sound Quality. Ph.D. Thesis.

[46] Krause C., Müller M., Wong H.W., Djamin V., Dutschke W., Obst U. (2014). Identification of bacteria in parenchymatous heartwood of living trees. Holzforschung.

[47] Guillemain P., Kronland-Martinet R., Ystad S. (1997). Physical modelling based on acoustic tubes of woodwind instrument bores exploiting wave variables. Acta Acust. United Acust..

[48] Shan Y., Junfeng W., Yue C., Jie L. (2018). Study on sound absorption performance of light wood wood. Res. Anal..

[49] Sakai H., Minamoto H., Mori T. (2011). Acoustic properties of wood used for the soundboards of musical instruments in relation to tree age. Holzforschung.

[50] Bucur V. (1995). Acoustics of Wood.

[51] Brémaud I., Gril J., Thibaut B. (2011). Anisotropy of wood vibrational properties: Dependence on grain angle and review of literature data. Wood Sci. Technol..

[52] Zou Y., Wang Z., Adjei P., Zhao X. (2023). The Sound Insulation Performance of Light Wood Frame Construction Floor Structure Based on Phononic Crystal Theory. J. Build. Eng..

[53] Darwish E.A., Midani M. (2023). The Potential of Date Palm Midribs-Based Fabric Acoustic Panels for Sustainable Interior Design. Ain Shams Eng. J..

[54] Piccardo C., Hughes M. (2022). Design Strategies to Increase the Reuse of Wood Materials in Buildings: Lessons from Architectural Practice. J. Clean. Prod..

[55] Caniato M., Marzi A., Silva S.M., Gasparella A. (2021). A Review of the Thermal and Acoustic Properties of Materials for Timber Building Construction. J. Build. Eng..

[56] Caniato M., Gasparella A., Bettarello F., Santoni A., Fausti P., Granzotto N., Bécot F.-X., Chevillotte F., Jaouen L., Borello G. (2022). A Reliability Study Concerning the Acoustic Simulations of Timber Elements for Buildings. Constr. Build. Mater..

[57] Nasir V., Ayanleye S., Kazemirad S., Sassani F., Adamopoulos S. (2022). Acoustic Emission Monitoring of Wood Materials and Timber Structures: A Critical Review. Constr. Build. Mater..

[58] Akiwate D.C., Date M.D., Venkatesham B., Suryakumar S. (2019). Acoustic Characterization of Additive Manufactured Perforated Panel Backed by Honeycomb Structure with Circular and Non-Circular Perforations. Appl. Acoust..

[59] Toyoda M., Tanak M., Takahashi D.J. (2007). Reduction of acoustic radiation by perforated board and honeycomb layer systems. Appl. Acoust..

[60] Sakagami K., Yamashita I., Yairi M., Minemura A. (2010). Sound absorption characteristics of a honeycomb—Backed microperforated panel absorber: Revised theory and experimental validation. Noise Control. Eng. J..

[61] Kuttruff H. (2009). Room Acoustics.

[62] (1998). Acoustics—Determination of Sound Absorption Coefficient and Impedance in Impedance Tubes, Part 2: Transfer-Function Method.

[63] Xu W.G., Jiang C.H., Zhang J.S. (2015). Improvement in underwater acoustic absorption performance of open-celled SiC foam. Colloids Surf. A Physicochem. Eng. Asp..

[64] Zou D.P., Willianms K.L., Thorsos E.I. (2015). Influence of temperature on acoustic sound speed and attenuation of seafloor sand sediment. IEEE J. Ocean. Eng..

[65] Arenas J.P., Crocker M.J. (2010). Recent Trends in Porous Sound-Absorbing Materials. Sound Vib..

[66] Randeberg R.T. (2000). Perforated Panel Absorbers with Viscous Energy Dissipation Enhanced by Orifice Design. Ph.D. Thesis.

[67] Kang J., Brocklesby M.W. (2005). Feasibility of applying microperforated absorbers in acoustic window systems. Appl. Acoust..

[68] Kang J., Fuchs H.V. (1999). Predicting the absorption of open weave textiles and micro-perforated membranes backed by an air space. J. Sound Vib..

[69] Kang J., Fuchs H.V. (1996). Predicting the absorption of porous materials backed by an air space. J. Sound Vib..

[70] Wu T.K. (2000). Boundary Element Acoustics: Fundamentals and Computer Codes.

[71] Liu J., Herrin D.W. (2010). Enhancing micro-perforated panel attenuation by partitioning the adjoining cavity. Appl. Acoust..

[72] Zhang Y., Wen J., Zhao X., Wen X., Ding Y. (2018). Theoretical analysis of multi-resonance sound absorption in multilayered microperforated panels. Appl. Acoust..

[73] Zhu X., Fan X., Liang B., Cheng J., Jing Y. (2017). Ultrathin acoustic metamaterial-based absorber with wide working bandwidth. Appl. Phys. A.

[74] Lee S.H., Park C.M., Seo Y.M., Wang Z.G., Kim C.K. (2009). Acoustic metamaterial with negative density. Appl. Phys. Lett..

[75] Zhang C., Luo C., Han J., Chen H., Yu D. (2017). Ultrathin acoustic metasurface-based Schroeder diffuser. Appl. Phys. Lett..

